# Retraction Note: Kindlin-2 promotes hepatocellular carcinoma invasion and metastasis by increasing Wnt/β-catenin signaling

**DOI:** 10.1186/s13046-025-03333-7

**Published:** 2025-02-28

**Authors:** Jie Lin, Wansong Lin, Yunbin Ye, Liping Wang, Xiaoyan Chen, Shengbing Zang, Aimin Huang

**Affiliations:** 1 Department of Pathology, The School of Basic Medical Sciences, Fujian Medical University, No.1, Xuefu North Road, University Town, Fuzhou, Fujian 350122 China; 2 Department of Pathology, Shengli Clinical Medical College of Fujian Medical University, Fuzhou, 350001 China; 3 Laboratory of Immuno-Oncology, Fujian Cancer Hospital and Fujian Medical University Cancer hospital, Fuzhou, 350014 China; 4 Fujian Provincial Key Laboratory of Translational Cancer Medicine, Fuzhou, 350014 China

**Retraction Note:** ***J Exp Clin Cancer Res*** **36, 134 (2017)**

10.1186/s13046-017-0603-4

The Editor-in-Chief has retracted this article. Following publication, the journal was alerted to potential mage integrity concerns, including:

- what appears to be a frameshift in Fig. 3c WT migration and invasion.
- an apparent overlap in Fig. 4, Fig. 5 and Fig. 6 MHCC97H 72kD, kindlin-2.
- an apparent overlap in Fig. 5 and Fig. 6 LM3/LV-sh 72kD, kindlin-2.
- apparent overlap Fig. 7b 0h LV-K2 + sictrl and LV-K2 + ICG-001.
- what appears to be a frameshiFig.  Fig. 7 LV-K2 + siβ-catenin and LV-K2 + ICG-001.

Therefore, the Editor has lost confidence in the data and conclusions of this article.

Authors Aimin Huang and Jie Lin have stated on behalf of all authors that they disagree with the retraction.

